# Safe use of the ketogenic diet in an infant with microcephaly, epilepsy, and diabetes syndrome: a case report

**DOI:** 10.1186/s12887-023-04272-y

**Published:** 2023-09-09

**Authors:** Walter A. Zegarra, William B. Gallentine, Maura R. Ruzhnikov, Catherine A. McAndrews, Anna L. Gloyn, Ananta Addala

**Affiliations:** 1 Division of Endocrinology, Department of Pediatrics, Stanford School of Medicine, Stanford University, Stanford, CA USA; 2 Department of Neurology and Neurological Sciences, Stanford School of Medicine, Stanford University, Stanford, CA USA; 3 Division of Medical Genetics, Department of Pediatrics, Stanford School of Medicine, Stanford University, Stanford, CA USA; 4 Stanford Diabetes Research Centre, Stanford School of Medicine, Stanford University, Stanford, CA USA; 5 Division of Pediatric Endocrinology, MC 5660, Center of Academic Medicine, 453 Quarry Road, Palo Alto, CA 94304 USA

**Keywords:** Ketogenic diet, Infantile spasms, Diabetic ketoacidosis, Genetics, Case report, Diabetic ketoacidosis

## Abstract

**Background:**

Microcephaly, epilepsy, and diabetes syndrome (MEDS) is a rare syndromic form of monogenic diabetes caused by bi-allelic loss of function mutations in *IER3IP1*. In vitro studies have shown that loss of *IER31P* leads to apoptosis in both neurons and pancreatic β-cells. Simultaneous management of seizures and diabetes is challenging in patients with MEDS. We present the challenges and successes in the use of ketogenic diet in an infant with insulinopenic diabetes.

**Case presentation:**

Our term female proband presented at 2 months of age with new onset multifocal seizures followed by the onset of infantile spasms (IS) at 4 months of age. An epilepsy gene panel identified bi-allelic variants, c.239T > G (p.Leu80*) and c.2T > A (initiator codon), in *IER3IP1* that were subsequently shown to be inherited in trans. Following initiation of steroid therapy for IS, the patient developed clinically apparent insulin requiring diabetes. Her epilepsy was ultimately refractory to multiple antiseizure medications, thus the ketogenic diet (KD) was initiated. We were able to successfully titrate to a therapeutic KD ratio of 3:1 and maintain a ketotic state without diabetic ketoacidosis (DKA). With intercurrent illnesses, however, the patient had rapid decompensation and mild DKA due to delays in treatment, and for this reason, KD was discontinued after 5 months.

**Conclusions:**

We report two novel *IER31P1* mutations in a patient with MEDS and the successful management of the cooccurring conditions of IS and insulinopenic diabetes with the KD. Our experience underscores the importance of careful monitoring during KD as our patient had DKA more easily when on the KD.

**Supplementary Information:**

The online version contains supplementary material available at 10.1186/s12887-023-04272-y.

## Background

 Microcephaly, epilepsy, and diabetes syndrome (MEDS) is also known as microcephaly with simplified gyral pattern, epilepsy, and permanent neonatal diabetes syndrome (OMIMID 614,231) [1, 2]. It is associated with congenital microcephaly with progressive head growth deceleration, a simplified gyral pattern on brain MRI, infantile onset of epilepsy including infantile spasms, refractory neonatal diabetes mellitus, and severe global developmental delays. MEDS is an autosomal recessive condition caused by pathogenic variants in the *IER3IP1* gene on chromosome 18q21 which encodes Immediate Early Response 3 Interacting Protein 1 (IER3IP1), a protein found in developing brain cortex and beta cells [2]. Bi-allelic loss of function variants in this gene lead to apoptosis in neurons and beta cells resulting in microcephaly, epilepsy, and insulinopenic diabetes [2]. MEDS has previously been described in 8 cases from Argentina, Morocco, and Egypt (Table 1) [1–4]. We present a proband with bi-allelic *IER3IP1* variants in trans and phenotypic features consistent with MEDS including microcephaly, insulinopenic diabetes mellitus, and a developmental and epileptic encephalopathy. Additionally, we report the use of both steroids and the ketogenic diet (KD) to ameliorate epilepsy symptoms, which presented an additional challenge in managing their insulin-dependent diabetes.

**Table 1:** Clinical manifestations of patients with MEDS reported to date

Case [Ref]	Mutation	Neurodevelopment	Seizure Type	Epilepsy onset	Diabetes onset	Diabetes control	Age of Death
1 [2]	c.62T>G (p.Val21Gly)	Severe PMD	Focal with secondary generalization and GTCS	2m	2m	Difficult	18m
2 [2]	c.233T>C (p.Leu78Pro)	N/A	GTCS, myoclonic	2m	At birth	Difficult	27m
3 [1]	c.233T>C (p.Leu78Pro)	Severe PMD	Tonic-clonic hemiconvulsion. Myoclonic and GTCS	Few days of life	<1wk	Difficult	5.5y
4 [1]	c.233T>C (p.Leu78Pro)	Severe PMD	Hypomotor seizures with abnormal eye deviation, myoclonic	2m	2m	Difficult	26m
5 [1]	c.233T>C (p.Leu78Pro)	PV/CH	Myoclonic	At birth	40d	N/A	3.5y
6 [1]	c.233T>C (p.Leu78Pro)	Normal	Hemiclonic with occasional eye and mouth twitches. Myoclonic.	2m	2wk	N/A	N/A
7 [3]	c.62T>G (p.Val21Gly)/ c.79delT (Phe27fsSer*25)	Severe PMD	Tonic-clonic, myoclonic	2m	2m	Good	8y
8 [4]	c.233T>C (p.Leu78Pro)	PV/CH	N/A	N/A	5wk	Difficult	7wk
9*	c.239T>G (p.Leu80*)/ c.2T>A (initiator codon)	PV/CH	Focal clonic, focal with impaired awareness. IS	2m	4m with steroid treatment	Good	N/A

*Abbreviations: N/A* Not available, *PMD* Psychomotor delay, *PV/CH* Poor visual responsiveness, central hypotonia, *GTCS* Generalized tonic-clonic seizures, *IS* Infantile spasms

^*^The case presented in this report

## Case presentation

**Fig. 1:**
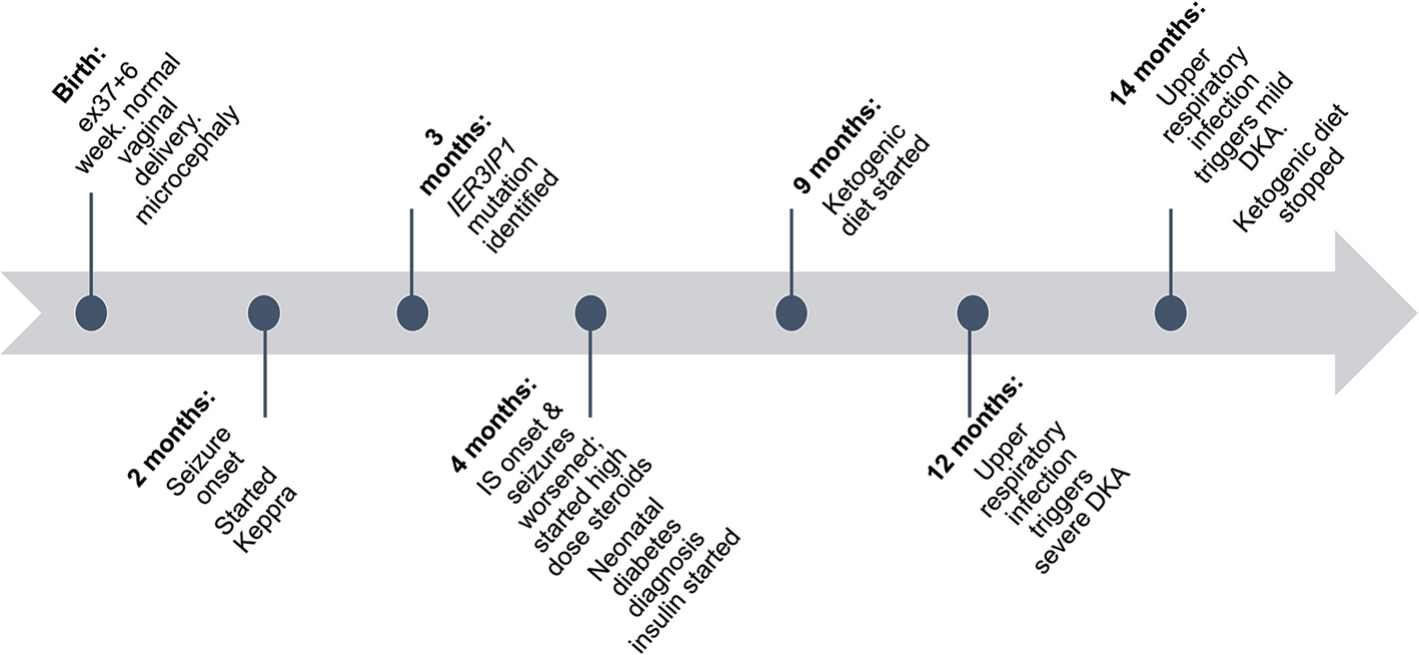
Clinical course of proband from birth to 14 months of life

The proband was born via normal spontaneous vaginal delivery at 37 weeks gestations after an uncomplicated prenatal history as well as pregnancy and delivery. She is the second-born to a non-consanguineous couple and her older brother is healthy and typically developing. At birth, growth parameters were normal (weight 2.715 kg, height 50 cm) except head size at 8th percentile (Fig. 1). Her newborn screening tests and development was normal for the first 2 months when she was taken to the emergency department for new onset seizures and found to have frequent multiple focal seizures on electroencephalogram (EEG). Magnetic resonance imaging revealed no parenchymal signal abnormalities but did demonstrate prominence of subarachnoid spaces and small bilateral subdural hygromas along the temporal convexities. Her seizures evolved to include infantile spasms as well as focal seizures and EEG was notable for diffuse encephalopathy, multiple focal epileptiform discharges, and hypsarrhythmia at the time of spasm onset – consistent with an epileptic encephalopathy. A multigene epilepsy panel was sent as part of her diagnostic work-up (Supplemental Table) reported two variants of uncertain significance in *IER3IP1 (NM_016097.4).* The first, c.239T > G (p.Leu80*), is novel and predicted to result in loss of function through protein truncation or haploinsufficiency due to nonsense-mediated decay. The second, c.2T > A (initiator codon), likely impacts transcription as it affects the start codon of the *IER3IP1* gene. Both variants meet the American College of Medical Genetics criteria for pathogenicity [5]. Sequencing the unaffected parents, obtained per clinical recommendations of the genetics team caring for the proband, demonstrated paternal inheritance for c.239T > G(p.Leu80*) and maternal inheritance for c.2T > A.

At 4 months of age, she was readmitted for infantile spasms and started high dose steroids. During this time, her blood glucose increased from 92 to 112 mg/dL before admission to 241-298 mg/dL at discharge. While on steroids, her parents reported an improvement in her general movements, eye tracking, and frequency of spasms. One week after discharge, steroids were discontinued due to persistent hyperglycemia (> 400 mg/dL) and despite multiple anti-seizure medications, seizures frequency, duration, and intensity increased. Thus, she was readmitted at 4.5 months of age to restart steroids and insulin initiation as her blood glucose levels increased to 200-400 mg/dL, consistent with beta cell dysfunction seen in MEDS. Blood glucose levels normalized on 0.35–0.7 units/kg/d of insulin treatment. Her blood glucose values were monitored with an intermittently scanned continuous glucose monitoring (isCGM).

### Ketogenic diet initiation

At 9 months old, she was admitted to initiate the KD for her treatment refractory epilepsy. Labs on admission (eight hours after last basal dose) demonstrated mild acidosis [beta-hydroxybutyrate (BHB) 0.6mmol/L, bicarbonate 17mmol/L, anion gap (AG) 15mmol/L]. The decision was made to advance the KD ratio slower than the standard protocol, starting with a ratio of 1:1. Given the low total daily dose of insulin (0.01 units/kilogram/day) at the time, we held her insulin. Blood glucose was evaluated every four hours and a chemistry panel assessing acidosis was measured every 12 h. The goal bicarbonate level was ≥ 15mmol/L, the diagnostic criteria for diabetic ketoacidosis (DKA) [6]. A safe BHB target was less clear due to lack of established guidelines in KD and insulinopenic diabetes, thus we set the initial goal BHB level at < 3mmol/L with careful observation [6].

**Fig. 2:**
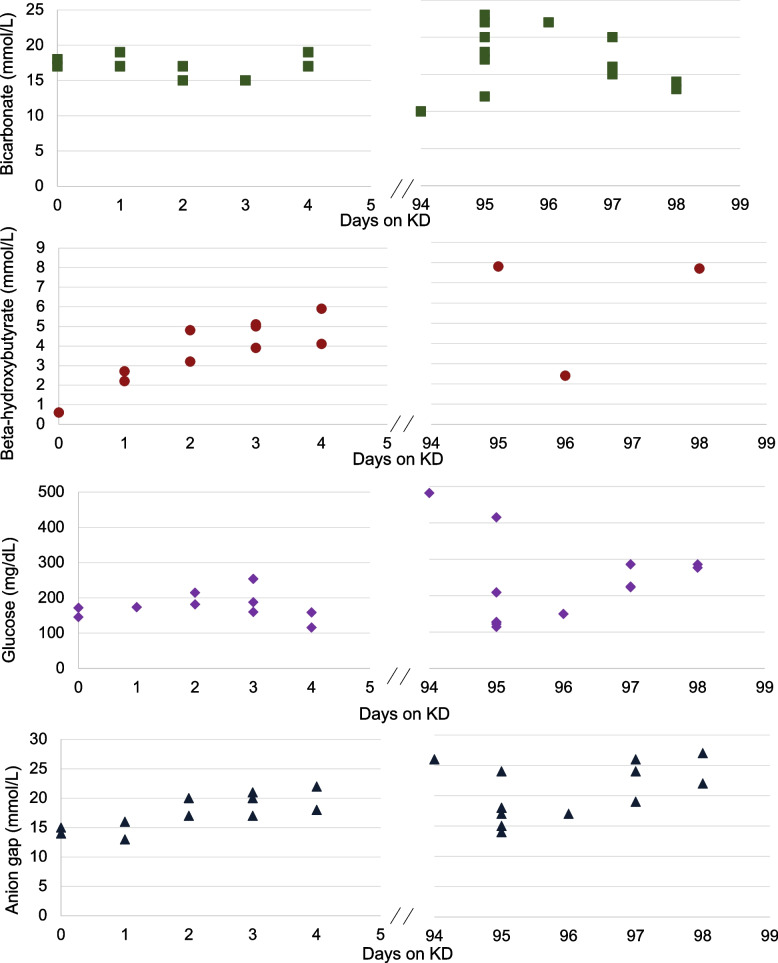
Biochemical monitoring while on ketogenic diet (KD). KD diet ratios: Day 1-ratio 1:1; Day 2–2:1; Day 3-2.5:1; Day 5 − 2:1; Day 30 − 3:1. The KD diet was weaned over a 4-week period decreasing the ketogenic ratio by 0.5:1 each week and was discontinued on day 98

By discharge, her KD ratio was optimized to 2.5:1 over the span of three days without significant change in her biochemistry (Fig. 2). She was restarted on insulin glargine 0.5 units nightly to facilitate a daily safety period to limit ketogenesis. Home monitoring included twice daily capillary ketone measurements and isCGM. Shortly after discharge, the ratio was decreased to 2:1 due hypoglycemia (60-70 mg/dL). One month after discharge, her insulin glargine was increased to 0.5 units twice daily and KD ratio increase to 3:1 due to hyperglycemia. With these changes, she had improvement in her responsiveness and engagement with her family and was euglycemic with ketones in the target range of 3–6 mmol/L.

Three months after KD initiation, she had a four-day episode of emesis, diarrhea, polyuria, hyperglycemia (isCGM 300-400 mg/dL), and increasing ketones (8-9mmol/L). The family contacted her pediatrician but were reassured because of high number of wet diapers. Due to the hyperglycemia, stepwise increase in insulin glargine was made. With a lack of response to the insulin changes and the progressive worsening of her symptoms (sunken eyes, chapped lips and clinical dehydration) she was taken to the emergency room. She was found to be in hyperosmolar DKA (pH 7.2, BUN 40 mg/dL, and Na 170mEq/L) and required significant fluid resuscitation and insulin drip with dextrose containing fluids. Attempts to preserve ketosis was not prioritized during this critical period. After her ketosis resolved, the family strongly favored restarting the diet (ratio of 3:1). One month after this episode, she had a second viral illness leading to DKA. Although the parents reported a clinical benefit from the KD, the diet was discontinued after these two episodes of DKA. The patient was weaned off the diet over a 4-week period decreasing the ratio by 0.5:1 each week.

At her most recent clinic visit, she continues to be euglycemic with insulin therapy and does not have profound acidosis with interim viral illnesses. She continues to have significant and refractory epileptic spasms and decreased alertness as before initiation of the KD.

## Discussion and conclusions

We present a case of an infant with MEDS due to two bi-allelic pathogenic variants in *IER3IP1* and refractory epileptic encephalopathy managed with steroids and the KD. Her management was complicated by a delicate balance between achieving ketosis for therapeutic purposes but avoiding severe ketosis which can result in DKA, cerebral edema, and other unwanted complications. In her clinical management, the difficulty is that the therapeutic effect of ketones for seizure prevention is fundamental in the pathway for causing DKA [7].

For ketone production (either therapeutic for the sake of seizure control or pathologic in the setting of DKA) there are three major mechanisms. First, decreasing carbohydrate intake and increasing fat intake results in fats being the primary fuel source (i.e., KD). Second, inducing a state of starvation requires utilization of metabolic stores (i.e., fat). Third, insulinopenic states result in an inability to inhibit lipolysis resulting in the production of ketones. Regardless of the mechanism of ketone production, ketosis with significant acidosis can lead to significant clinical complications including cerebral edema. At the time of KD initiation, our patient had a very low insulin requirement of only 0.1 units/kg/day. Given this low insulin requirement paired with the requisite limits to carbohydrates of the KD, she was considered to be at risk for euglycemic DKA. Furthermore, initiation and maintenance of therapeutic KD requires the production of ketones, and therefore, some level of initial acidosis.

One of the most challenging aspects of initiating the KD in the setting insulinopenic diabetes in our case was identifying safe biochemical ranges. While we use standard bicarbonate cutoff for DKA to set bicarbonate goals, the goal hydroxybutyrate level was much less clear because of the patient’s unique physiology and pathophysiology. For this reason, we opted to start with targets informed by prior case reports. Ultimately, we found that the patient was safe when her capillary ketones were ≤6mmol/L, higher than our initial anticipated goal for an infant with insulinopenic diabetes but the standard range for KD. We also demonstrated that despite the use of insulin, which prevents lipolysis and ketogenesis, steady state ketosis can be achieved in an infant on KD. To ensure safety, we found that isCGM/CGM and twice daily capillary ketone monitoring provided adequate triggers to escalate care.

For this patient, the distance from our hospital was a major limiting factor in receiving prompt care for her decompensating episodes and this was ultimately the reason for discontinuation of the KD. Her course also demonstrates an anticipated consequence of the KD in a person with insulinopenic diabetes: the threshold for DKA is much lower and should be a serious consideration in the initiation of the KD in persons with insulinopenic diabetes. Ethically, this was an important point of discussion with the family, particularly given that the cause of major morbidity and mortality in infants with *IER31P1* mutations is respiratory illness and decompensation which maybe compounded by DKA.

We report a proband with two new pathogenic variants of the *IER3IP1* gene causing MEDS requiring management with KD in the setting of insulinopenic diabetes. We found that, despite the use of insulin, steady state ketosis can be achieved in an infant with a KD. To assure safety, we found that CGM to monitor for hyperglycemia or hypoglycemia, and twice daily capillary ketone monitoring provided adequate triggers to escalate care. The threshold for DKA is much lower and should be a serious consideration in the initiation of a KD in persons with insulinopenic diabetes. This should be discussed, and anticipatory guidance should be in place with the family and care team, including the primary pediatrician.

### Supplementary Information

**Additional file 1:** **Supplementary Table 1. **Genes included in the invitae epilepsy panel.

## Data Availability

The datasets generated and/or analysed during the current study were executed via clinical testing through Invitae Epilepsy Panel.

## Abbreviations

MEDS: Microcephaly, epilepsy, and diabetes syndrome
IERI3P1: Immediate Early Response 3 Interacting Protein 1
IS: Infantile spasms
KD: Ketogenic diet
DKA: Diabetic ketoacidosis
EEG: Electroencephalogram
isCGM: intermittently scanned continuous glucose monitoring
BHB: beta-hydroxybutyrate
AG: anion gap
PMD: Psychomotor delay
PV/CH: Poor visual responsiveness, central hypotonia
GTCS: Generalized tonic-clonic seizures
