# Supplementary material for: Safe use of the ketogenic diet in an infant with microcephaly, epilepsy, and diabetes syndrome: a case report
Source: BMC Pediatr. 2023 Sep 9;23:453. doi: 10.1186/s12887-023-04272-y (PMC10492392; doi:10.1186/s12887-023-04272-y)
Supplement: Supplementary file 1 — Additional file 1: Supplementary Table 1. Genes included in the invitae epilepsy panel. [file 12887_2023_4272_MOESM1_ESM.docx]

**Supplementary Table 1: Genes included in the Invitae Epilepsy Panel**

| *AARS* | *ABAT* | *ADAR* | *ADSL* | *ALDH5A1* | *ALDH7A1* | *ALG1* | *ALG12* |
| --- | --- | --- | --- | --- | --- | --- | --- |
| *ALG13* | *ALG6* | *AMACR* | *AMT* | *AP2M1* | *AP3B2* | *ARG1* | *ARHGEF15* |
| *ARHGEF9* | *ARSA* | *ARX* | *ASAH1* | *ASNS* | *ATAD1* | *ATP1A2* | *ATP1A3* |
| *ATP6AP2* | *ATRX* | *BRAT1* | *C12orf57* | *CACNA1A* | *CACNA1E* | *CACNA1H* | *CACNA2D2* |
| *CAD* | *CAMK2B* | *CARS2* | *CASK* | *CCDC88A* | *CDKL5* | *CERS1* | *CHD2* |
| *CHRNA2* | *CHRNA4* | *CHRNB2* | *CLCN4* | *CLCN6* | *CLN2 (TPP1)* | *CLN3* | *CLN5* |
| *CLN6* | *CLN8* | *CLTC* | *CNTN2* | *CNTNAP2* | *COG5* | *COL18A1* | *CSTB* |
| *CTNNB1* | *CTSD* | *CYFIP2* | *CYP27A1* | *DDC* | *DDX3X* | *DEAF1* | *DEPDC5* |
| *DHDDS* | *DHFR* | *DIAPH1* | *DNAJC5* | *DNM1* | *DNM1L* | *DOCK7* | *DYNC1H1* |
| *DYRK1A* | *ECHS1* | *EEF1A2* | *EHMT1* | *EMC1* | *EPM2A* | *FAR1* | *FARS2* |
| *FASN* | *FBXO11* | *FGF12* | *FOLR1* | *FOXG1* | *FRRS1L* | *GABBR2* | *GABRA1* |
| *GABRB1* | *GABRB2* | *GABRB3* | *GABRG2* | *GAMT* | *GATAD2B* | *GATM* | *GCH1* |
| *GLDC* | *GLRA1* | *GLRB* | *GNAO1* | *GNB1* | *GOSR2* | *GPAA1* | *GPHN* |
| *GRIA3* | *GRIN1* | *GRIN2A* | *GRIN2B* | *GRIN2D* | *GTPBP3* | *GUF1* | *HCN1* |
| *HEXA* | *HNRNPU* | *HTT* | *IDH3A* | *IER3IP1* | *IFIH1* | *IQSEC2* | *ITPA* |
| *KANSL1* | *KCNA1* | *KCNA2* | *KCNB1* | *KCNC1* | *KCND2* | *KCNH1* | *KCNH2* |
| *KCNH5* | *KCNJ10* | *KCNK4* | *KCNMA1* | *KCNQ2* | *KCNQ3* | *KCNQ5* | *KCNT1* |
| *KCTD7* | *KIF1A* | *KIF2A* | *KIF5A* | *KPNA7* | *LAMC3* | *LGI1* | *LIAS* |
| *LMNB2* | *MBD5* | *MDH2* | *MECP2* | *MEF2C* | *MFSD8* | *MOCS1* | *MOCS2* |
| *MOCS3* | *MTOR* | *NACC1* | *NAGLU* | *NECAP1* | *NEDD4L* | *NEXMIF* | *NGLY1* |
| *NHLRC1* | *NPC1* | *NPC2* | *NPRL3* | *NRXN1* | *NTRK2* | *NUS1* | *PACS1* |
| *PACS2* | *PAFAH1B1* | *PCDH19* | *PCLO* | *PEX10* | *PEX12* | *PEX13* | *PEX14* |
| *PEX16* | *PEX19* | *PEX2* | *PEX26* | *PEX3* | *PEX5* | *PEX6* | *PHGDH* |
| *PIGA* | *PIGG* | *PIGN* | *PIGO* | *PIGP* | *PIGQ* | *PIGV* | *PIGW* |
| *PIK3AP1* | *PLAA* | *PLCB1* | *PNKD* | *PNKP* | *PNPO* | *PNPT1* | *POLG* |
| *PPP2CA* | *PPP2R1A* | *PPP2R5D* | *PPP3CA* | *PPT1* | *PRDM8* | *PRICKLE1* | *PRICKLE2* |
| *PRIMA1* | *PRRT2* | *PSAP* | *PSAT1* | *PSPH* | *PTPN23* | *PURA* | *QARS* |
| *QDPR* | *RAB11A* | *RAB11B* | *RAI1* | *RALA* | *RANBP2* | *RBFOX1* | *RBFOX3* |
| *RELN* | *RFT1* | *RHOBTB2* | *RNASEH2A* | *RNASEH2B* | *RNASEH2C* | *RNF13* | *ROGDI* |
| *RORB* | *RUSC2* | *SAMHD1* | *SATB2* | *SCARB2* | *SCN1A* | *SCN1B* | *SCN2A* |
| *SCN3A* | *SCN5A* | *SCN8A* | *SCN9A* | *SCP2* | *SERPINI1* | *SETBP1* | *SGCE* |
| *SGSH* | *SIK1* | *SLC12A5* | *SLC13A5* | *SLC19A3* | *SLC1A2* | *SLC25A12* | *SLC25A22* |
| *SLC2A1* | *SLC35A2* | *SLC6A1* | *SLC6A5* | *SLC6A8* | *SLC9A6* | *SMC1A* | *SNAP25* |
| *SNIP1* | *SNX27* | *SPATA5* | *SPTAN1* | *ST3GAL3* | *ST3GAL5* | *STAG2* | *STRADA* |
| *STX1B* | *STXBP1* | *STXBP2* | *SUMF1* | *SUOX* | *SYN1* | *SYNGAP1* | *SYNJ1* |
| *SZT2* | *TANGO2* | *TBC1D24* | *TBCK* | *TBL1XR1* | *TCF4* | *TH* | *TK2* |
| *TPK1* | *TREX1* | *TSC1* | *TSC2* | *TSFM* | *TUBA8* | *TUBB2A* | *UBA5* |
| *UBE3A* | *UNC80* | *WDR45* | *WWOX* | *YWHAG* | *ZDHHC9* | *ZEB2* | *ZSWIM6* |
